# Event-Triggering State and Fault Estimation for a Class of Nonlinear Systems Subject to Sensor Saturations

**DOI:** 10.3390/s21041242

**Published:** 2021-02-10

**Authors:** Cong Huang, Bo Shen, Lei Zou, Yuxuan Shen

**Affiliations:** 1 College of Information Science and Technology, Donghua University, Shanghai 201620, China; c.huang@mail.dhu.edu.cn; 2 Engineering Research Center of Digitalized Textile and Fashion Technology, Ministry of Education, Shanghai 201620, China; 3 College of Electrical Engineering and Automation, Shandong University of Science and Technology, Qingdao 266590, China; zouleicup@gmail.com; 4 Artificial Intelligence Energy Research Institute, Northeast Petroleum University, Daqing 163318, China; shenyuxuan5973@163.com; 5 Heilongjiang Provincial Key Laboratory of Networking and Intelligent Control, Northeast Petroleum University, Daqing 163318, China

**Keywords:** event-triggering mechanism (ETM), nonlinear system, recursive estimator, sensor saturations, state and fault estimation

## Abstract

This paper is concerned with the state and fault estimation issue for nonlinear systems with sensor saturations and fault signals. For the sake of avoiding the communication burden, an event-triggering protocol is utilized to govern the transmission frequency of the measurements from the sensor to its corresponding recursive estimator. Under the event-triggering mechanism (ETM), the current transmission is released only when the relative error of measurements is bigger than a prescribed threshold. The objective of this paper is to design an event-triggering recursive state and fault estimator such that the estimation error covariances for the state and fault are both guaranteed with upper bounds and subsequently derive the gain matrices minimizing such upper bounds, relying on the solutions to a set of difference equations. Finally, two experimental examples are given to validate the effectiveness of the designed algorithm.

## 1. Introduction

State estimation/filtering problems have always been one of the fundamental issues in the areas of target tracking, navigation and positioning, electric power systems, econometrics, biosystems, etc. Therefore, enormous research attention has been paid to the state estimation problems and some elegant work has been reported, see e.g., [1,2,3,4,5]. According to different performance indices, the current state estimation approaches include Kalman filtering (KF), extend Kalman filtering (EKF), $H_{\infty }$ filtering and so on. To be specific, the famous KF approach has been proposed in [2] by providing optimal state estimates in the sense of minimal mean-squared error under the assumption that system parameters and noise statistics are precisely known. The $H_{\infty }$ filtering method proposed [6,7] is capable to attenuate the influence from the exogenous disturbance to the filtering error. When it comes to the case that the system model is nonlinear or uncertain, the celebrated EKF approach has been shown to be a useful tool for the state estimation issues. For instance, in [8], the EKF approach has been developed to cope with the nonlinear systems subject to missing measurement. Moreover, in [9], the filtering approach has also been applied in the complex networks with incomplete measurements.

It is often the case that the faults are inevitable in practical applications because of a variety of reasons including component failure, ageing equipment, complex external environment, bandwidth limitation, etc. During the past few decades, fault detection and fault-tolerant control issues have gained considerable research enthusiasm due to the demand in reliability and security of the practical systems. It should be pointed out that the accurate information of the fault signals are hard to acquire, whilst the recently emerging state and fault estimation (SFE) approach provides a good solution to obtain the sufficient information of the state and fault signals simultaneously. By such a merit, increasing research attention has been paid on this aspect recently, and some inspiring work has been available in the literature [10,11,12]. For instance, an SFE algorithm relying on a recursive approach has been designed in [13] for uncertain systems with missing measurements and stochastic nonlinearities. $H_{\infty }$ SFE problems have also been studied for various dynamic systems, such as fuzzy systems [12,14], nonlinear systems [15,16] and 2-D systems [17,18]. Nevertheless, the SFE problems have not been thoroughly investigated yet and still have been a research hotspot in control/filtering community.

In reality, sensors may not always provide signals with unlimited amplitudes owing to the physical constraints. If the sensor saturation is not properly handled, it will severely decrease the system performance. The main challenge of research on the saturations in control community is how to design a filtering/control algorithm that can effectively dealing with the nonlinearities brought by the sensor saturations. As a consequence, the filtering/control issues subject to sensor saturations have gained initial research focus, see, e.g., [19,20]. For example, a recursive filtering issue has been solved for uncertain systems with faults and sensor saturations in [21]. In [22,23], the $H_{\infty }$ filtering issues have been settled for nonlinear systems with incomplete measurements and sensor saturations. In [20], mean-squared consensus control problem has been studied for stochastic multi-agent systems subject to sensor saturations where the desired controllers have been designed depending on the solutions to recursive matrix inequalities.

On another research front, the event-triggering mechanism (ETM) has become a research hotspot recently due mainly to its superiority of effectively reducing communication resources compared with the traditional time-triggering protocol. Under the ETM the current measurement will be transmitted only when the predefined triggering condition is met, and thereby the transmission numbers can be reduced largely. Based on this idea, various control and filtering issues under the ETM have been studied, see e.g., [6,24,25,26]. Very recently, considerable research attention has been paid on the event-triggering fault estimation issue owing to its vital role in the practical engineering. Accordingly, the event-triggering fault estimation problems have been investigated for various systems, such as nonlinear systems with missing measurements [27], stochastic systems subject to nonlinearities and packet dropouts [28], and stochastic systems with deception attacks [29]. However, to the best of the authors’ knowledge, the event-triggering state and fault estimation (ETSFE) problem for nonlinear systems with sensor saturations has not been fully studied, which constitutes the main motivation of this paper.

In terms of the methodologies, due to the effects brought by nonlinearities (including the saturation functions) and event-triggering protocol, it is almost impossible in the Kalman filtering framework to minimize the estimation error covariance through adjustment of the gain matrices. In [30], an alternative way has been proposed to handle the effects of norm bounded parameter uncertainties and a robust filter has been designed such that an upper bounded matrix of the estimation error covariance is minimized. Enlightened by this idea, such a filtering approach has been applied in various complex systems such as complex networks [9] and sensor networks [31]. However, it should be pointed out that, for the state and fault simultaneous estimation problem in the existing literature, the estimation error covariance minimization method has still been the main method which is incapable of dealing with more real complex phenomena. Therefore, it is the second motivation of this paper to develop the filtering approach proposed in [30] to handle the state and fault estimation problems with sensor saturations under the event-triggering strategy.

The novelties of this paper are emphasized as follows: (1) a novel ETSFE issue is, for the first time, addressed when the effects of sensor saturations, nonlinearities as well as ETM are simultaneously taken into consideration; (2) the state and fault estimator is designed such that the upper bounds on the error covariances of the state and fault estimation are respectively guaranteed at each time instant; and (3) the gain matrices are designed via two recursions which minimize the obtained upper bounds. Finally, two illustrative examples are utilized to verify the feasibility of the developed ETSFE algorithm.

The remaining part of this paper is organized as follows. In Section 2, the problem to be investigated is addressed. The main results are listed in Section 3 where the desired state and fault estimators is designed. In Section 4, two illustrative examples are given and the conclusion is drawn in Section 5.

**Notations**: In this paper, the notations mentioned are standard. $R^{n}$ and $R^{m\times n}$ respectively denote the *n*-dimensional Euclidean space and m×n real matrix. *I* is the identity matrix, while $\mathrm{diag}\{a_{1},a_{2},\ldots ,a_{N}\}$ represents the block-diagonal matrix with matrices $a_{1},a_{2},\cdots ,a_{N}$. For symmetric matrices *x* and *y*, x≥y(x>y) means that x−y is positive semi-define (positive definite) matrix. The superscript “*T*” and “−1” refer to matrix transposition and inverse, respectively. R(M) is the rank of the matrix *M*. E{x} denotes the mathematical expectation of the stochastic variable *x*. tr{M} denotes the trace of the matrix *M*. ∥·∥ stands for the Euclidean norm.

## 2. Problem Formulation

**Figure 1:**
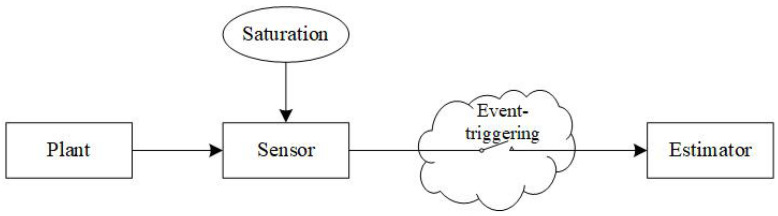
Structure of the state and fault estimation.

The estimation structure under consideration is shown in Figure 1 and the dynamics of the plant is given by

(1) $$x_{k+1}=h_{k}\left(x_{k}\right)+B_{k}f_{k}+w_{k}$$

where $x_{k}\in R^{n_{x}}$ is the system state vector, $f_{k}\in R^{n_{f}}$ represents a fault signal, $w_{k}\in R^{n_{x}}$ is the process noise, and $B_{k}$ is a given compatible matrix.

The measurements with sensor saturation are described by

(2) $$y_{k}=\vartheta \left(C_{k}x_{k}\right)+D_{k}f_{k}+v_{k}$$

where $y_{k}\in R^{n_{y}}$ represents the measurement vector at time instant *k*, $v_{k}\in R^{n_{y}}$ is the measurement noise, and $C_{k}$, $D_{k}$ are both appropriate-dimensional matrices.

**Assumption** **1.** *The matrix $D_{k}$ is full column rank, i.e., $R\left(D_{k}\right)=n_{f}$, $n_{f}\leq n_{y}$.*

The nonlinear function $h_{k}\left(\cdot \right)$ satisfies the following condition

(3) $$∥h_{k}\left(X\right)-h_{k}\left(Y\right)∥\leq \nu_{k}∥X-Y∥,\;\forall \;\;X,\;Y\in R^{n_{x}}$$

where $\nu_{k}>0$ is a known matrix.

The noise signals $w_{k}$ and $v_{k}$ have the following statistical properties

(4) $$\begin{array}{cc} \; & E\left\{w_{k}\right\}=0,\;E\left\{w_{k}w_{l}^{T}\right\}=R_{k}\delta_{kl},\\ \; & E\left\{v_{k}\right\}=0,\;E\left\{v_{k}{v_{l}}^{T}\right\}=Q_{k}\delta_{kl} \end{array}$$

where $R_{k}>0$ and $Q_{k}>0$ are known appropriate-dimensional matrices, and $\delta_{kl}$ represents the Kronecker function with

$$\delta_{kl}=\left\{\begin{array}{c} 1,\;k=l,\\ 0,\;k\neq l. \end{array}\right.$$

The saturation function ϑ(·): $R^{n_{y}}$↦$R^{n_{y}}$ is defined by

(5) $$\vartheta \left(s\right)={\left[\vartheta \left(s_{1}\right)\;\vartheta \left(s_{2}\right)\;\cdots \;\vartheta \left(s_{n_{y}}\right)\right]}^{T}$$

with $\vartheta (s_{i})$ = $\mathrm{sign}\left(s_{i}\right)\;\min\{\varrho_{i},|s_{i}\left|\right\}$$\left(i=1,2,\ldots ,n_{y}\right)$, where $s_{i}$ is the *i*th element of vector *s*, sign(·) represents the signum function, and $\varrho_{i}$ denotes the saturation level for *i*th element.

For the sake of reducing limited communication resource, the ETM is adopted to govern the transmission frequency between the sensor and the estimator. We denote the transmission instants by $0=k_{0}<k_{1}<k_{2}<\cdots <k_{l}<\cdots$, which is determined by

(6) $$\begin{array}{cc} k_{l+1}= & \min\left\{k\in N|k>k_{l},\;∥y_{k}-y_{k_{l}}∥>\tau \right\} \end{array}$$

where τ>0 is a given scalar and $y_{k_{l}}$ is the measurement transmitted at the latest time.

For the purpose of estimating the state and fault simultaneously, we construct the estimator as follows

(7) $$\left\{\begin{array}{cc} \; & {\hat{x}}_{k+1}=h_{k}\left({\hat{x}}_{k}\right)+B_{k}{\hat{f}}_{k}+G_{k}\left(y_{k_{l}}-C_{k}{\hat{x}}_{k}-D_{k}{\hat{f}}_{k}\right)\\ \; & {\hat{f}}_{k}=L_{k}\left(y_{k_{l}}-C_{k}{\hat{x}}_{k}\right) \end{array}\right.$$

where ${\hat{f}}_{k}$, ${\hat{x}}_{k}$ represent the estimates of fault and state respectively and $L_{k}$, $G_{k}$ are the estimator parameters respectively.

Let the state estimation error and fault estimation error be ${\tilde{x}}_{k}=x_{k}-{\hat{x}}_{k}$ and ${\tilde{f}}_{k}=f_{k}-{\hat{f}}_{k}$, respectively.

By noting (1) and (7), one has

(8) $$\begin{array}{cc} {\tilde{x}}_{k+1}= & h_{k}\left(x_{k}\right)-h_{k}\left({\hat{x}}_{k}\right)+\left(B_{k}-G_{k}D_{k}\right){\tilde{f}}_{k}+G_{k}\varepsilon_{k}\\ \; & -G_{k}\vartheta \left(C_{k}x_{k}\right)-G_{k}v_{k}+G_{k}C_{k}{\hat{x}}_{k}+w_{k} \end{array}$$

and

(9) $${\tilde{f}}_{k}=f_{k}-L_{k}D_{k}f_{k}-L_{k}\left(\vartheta \left(C_{k}x_{k}\right)+v_{k}-\varepsilon_{k}-C_{k}{\hat{x}}_{k}\right)$$

where $\varepsilon_{k}=y_{k}-y_{k_{l}}$.

Assuming that the constraint condition $L_{k}D_{k}=I$ is met, we eventually derive

(10) $${\tilde{f}}_{k}=-L_{k}\left(\vartheta \left(C_{k}x_{k}\right)+v_{k}-\varepsilon_{k}-C_{k}{\hat{x}}_{k}\right).$$

**Remark** **1.** *The constraint condition $L_{k}D_{k}=I$ plays a key role in the estimator design. It is obvious that the fault estimator (9) contains the fault vectors $f_{k}$. Since the dynamics of the faults is generally unknown, the fault term should be eliminated. To this end, $L_{k}D_{k}=I$ is introduced as an additional condition.*

Then, we define the estimation error covariances of the state and fault as follows

(11) $$P_{k}^{x}=E\left\{{\tilde{x}}_{k}{\tilde{x}}_{k}^{T}\right\},\;P_{k}^{f}=E\left\{{\tilde{f}}_{k}{\tilde{f}}_{k}^{T}\right\}.$$

Our main objective of this paper is to develop an event-triggering state and fault estimator of the form (7) such that, for all nonlinearities as well as sensor saturations, the upper bounds $\left(\Sigma_{k}^{x}\;\mathrm{and}\;\Sigma_{k}^{f}\right)$ for the estimation error covariances of state and fault are respectively guaranteed, that is

(12) $$\begin{array}{cc} P_{k}^{x}= & E\left\{{\tilde{x}}_{k}{\tilde{x}}_{k}^{T}\right\}\leq \Sigma_{k}^{x},\\ P_{k}^{f}= & E\left\{{\tilde{f}}_{k}{\tilde{f}}_{k}^{T}\right\}\leq \Sigma_{k}^{f}. \end{array}$$

Moreover, the designed gain matrices $G_{k}$ and $L_{k}$ are expected to minimize the upper bound $\Sigma_{k}^{x}$ and $\Sigma_{k}^{f}$ simultaneously at each iteration.

## 3. Main Results

In this section, the upper bounds on the estimation error covariances of the state and fault are expressed by means of recursions. Then, the proper gain matrices $G_{k}$ and $L_{k}$ are designed to minimize the upper bounds on the estimation error covariances and fault error covariances, respectively. The following lemmas will be used for obtaining the results.

**Lemma** **1 ([4]).** *For ∀k∈[0,N], let the matrix function be $\Xi_{k}\left(\cdot \right):R^{n\times n}\mapsto R^{n\times n}$, and arbitrary symmetric matrices x>0 and y>0. If $\Xi_{k}\left(x\right)\leq \Xi_{k}\left(y\right)$ for all x≤y, then under the initial condition $G_{0}=H_{0}$, the solutions $G_{k}$ and $H_{k}$ to difference equations $G_{k+1}=\Xi_{k}\left(G_{k}\right),$ and $H_{k+1}=\Xi_{k}\left(H_{k}\right)$ satisfy $G_{k+1}\leq H_{k+1}$.*

**Lemma** **2 ([8]).** *The following relationship is true for arbitrary real vectors M and N*

$$MN^{T}+MN^{T}\leq \epsilon MM^{T}+\epsilon^{-1}NN^{T}$$

*where ϵ>0 is an arbitrary scalar.*

**Lemma** **3.** *Under the constraint condition $L_{k}D_{k}=I$, the fault error covariance $P_{k}^{f}=E\left\{{\tilde{f}}_{k}{\tilde{f}}_{k}^{T}\right\}$ can be derived as follows*

(13) $$P_{k}^{f}=L_{k}\Lambda_{k}L_{k}^{T}$$

*where*

$$\begin{array}{cc} \Lambda_{k}= & E\left\{\vartheta \left(C_{k}x_{k}\right)\vartheta^{T}\left(C_{k}x_{k}\right)+C_{k}{\hat{x}}_{k}{\hat{x}}_{k}^{T}C_{k}^{T}+\varepsilon_{k}\varepsilon_{k}^{T}+\varepsilon_{k}{\hat{x}}_{k}^{T}C_{k}^{T}\right.\\ \; & +\left.C_{k}{\hat{x}}_{k}\varepsilon_{k}^{T}-\vartheta \left(C_{k}x_{k}\right)\varepsilon_{k}^{T}-\varepsilon_{k}\vartheta^{T}\left(C_{k}x_{k}\right)-C_{k}{\hat{x}}_{k}\vartheta^{T}\left(C_{k}x_{k}\right)\right.\\ \; & +\left.Q_{k}-\vartheta \left(C_{k}x_{k}\right){\hat{x}}_{k}^{T}C_{k}^{T}-\varepsilon_{k}v_{k}^{T}-v_{k}\varepsilon_{k}^{T}\right\}. \end{array}$$

**Proof.**  The validation of (13) can be verified by noting (10) and (11) and the rest of proof is omitted. □

**Lemma** **4.** *Under the condition $L_{k}D_{k}=I$, the state estimation error covariance $P_{k}^{x}$=$E\{{\tilde{x}}_{k}{\tilde{x}}_{k}^{T}\}$ is derived by*

(14) $$\begin{array}{cc} P_{k+1}^{x}= & E\left\{\left(h_{k}\left(x_{k}\right)-h_{k}\left({\hat{x}}_{k}\right)\right){\left(h_{k}\left(x_{k}\right)-h_{k}\left({\hat{x}}_{k}\right)\right)}^{T}+\left(B_{k}-G_{k}D_{k}\right){\tilde{f}}_{k}{\tilde{f}}_{k}^{T}{\left(B_{k}-G_{k}D_{k}\right)}^{T}\right.\\ \; & \left.+G_{k}\vartheta \left(C_{k}x_{k}\right)\vartheta^{T}\left(C_{k}x_{k}\right)G_{k}^{T}+G_{k}\varepsilon_{k}\varepsilon_{k}^{T}G_{k}^{T}+G_{k}C_{k}{\hat{x}}_{k}{\hat{x}}_{k}^{T}C_{k}^{T}G_{k}^{T}\right\}+R_{k}+G_{k}Q_{k}G_{k}^{T}\\ \; & -{ℜ}_{k,1}-{ℜ}_{k,1}^{T}+{ℜ}_{k,2}+{ℜ}_{k,2}^{T}+{ℜ}_{k,3}+{ℜ}_{k,3}^{T}+{ℜ}_{k,4}+{ℜ}_{k,4}^{T}+{ℜ}_{k,5}+{ℜ}_{k,5}^{T}+{ℜ}_{k,6}+{ℜ}_{k,6}^{T}\\ \; & -{ℜ}_{k,7}-{ℜ}_{k,7}^{T}-{ℜ}_{k,8}-{ℜ}_{k,8}^{T}-{ℜ}_{k,9}-{ℜ}_{k,9}^{T}+{ℜ}_{k,10}+{ℜ}_{k,10}^{T}-{ℜ}_{k,11}-{ℜ}_{k,11}^{T} \end{array}$$

*where*

$$\begin{array}{cc} {ℜ}_{k,1}= & E\left\{\left(h_{k}\left(x_{k}\right)-h_{k}\left({\hat{x}}_{k}\right)\right)\vartheta^{T}\left(C_{k}x_{k}\right)\right\}G_{k}^{T},\;{ℜ}_{k,2}=E\left\{\left(h_{k}\left(x_{k}\right)-h_{k}\left({\hat{x}}_{k}\right)\right){\tilde{f}}_{k}^{T}\right\}{\left(B_{k}-G_{k}D_{k}\right)}^{T},\\ {ℜ}_{k,3}= & E\left\{\left(h_{k}\left(x_{k}\right)-h_{k}\left({\hat{x}}_{k}\right)\right){\hat{x}}_{k}^{T}\right\}C_{k}^{T}G_{k}^{T},\;{ℜ}_{k,4}=E\left\{\left(h_{k}\left(x_{k}\right)-h_{k}\left({\hat{x}}_{k}\right)\right)\varepsilon_{k}^{T}\right\}G_{k}^{T},\\ {ℜ}_{k,5}= & \left(B_{k}-G_{k}D_{k}\right)E\left\{{\tilde{f}}_{k}{\hat{x}}_{k}^{T}\right\}C_{k}^{T}G_{k}^{T},\;{ℜ}_{k,6}=\left(B_{k}-G_{k}D_{k}\right)E\left\{{\tilde{f}}_{k}\varepsilon_{k}^{T}\right\}G_{k}^{T},\\ {ℜ}_{k,7}= & \left(B_{k}-G_{k}D_{k}\right)E\left\{{\tilde{f}}_{k}\vartheta^{T}\left(C_{k}x_{k}\right)\right\}G_{k}^{T},\;{ℜ}_{k,8}=G_{k}E\left\{\vartheta \left(C_{k}x_{k}\right){\hat{x}}_{k}^{T}\right\}C_{k}^{T}G_{k}^{T},\\ {ℜ}_{k,9}= & G_{k}E\left\{\vartheta \left(C_{k}x_{k}\right)\varepsilon_{k}^{T}\right\}G_{k}^{T},\;{ℜ}_{k,10}=G_{k}C_{k}E\left\{{\hat{x}}_{k}\varepsilon_{k}^{T}\right\}G_{k}^{T},\;{ℜ}_{k,11}=G_{k}v_{k}\varepsilon_{k}^{T}G_{k}^{T}. \end{array}$$

**Proof.**  It can be shown that (14) follows directly from (8) and (11), and the proof is omitted for conciseness. □

### 3.1. Fault Estimation

The following Theorem 1 provides the explicit form of the upper bound on the fault estimation error covariance $P_{k}^{f}$ in terms of the recursion.

**Theorem** **1.** *Consider the fault estimation error covariance in (13). Assume that the condition $L_{k}D_{k}=I$ is satisfied. For any given positive scalars $a_{k}$, $b_{k}$, $c_{k}$ and $d_{k}$, the upper bound on the fault estimation error covariance $P_{k}^{f}$ is obtained by*

(15) $$\Sigma_{k}^{f}=L_{k}{\bar{\Lambda }}_{k}L_{k}^{T}$$

*where*

(16) $$\begin{array}{cc} {\bar{\Lambda }}_{k}= & \kappa_{1}\tau^{2}I+\kappa_{2}\sum_{i=1}^{n_{y}}\varrho_{i}^{2}I+\kappa_{3}C_{k}{\hat{x}}_{k}{\hat{x}}_{k}^{T}C_{k}^{T}+\kappa_{4}Q_{k},\\ \kappa_{1}= & 1+a_{k}^{-1}+b_{k}+d_{k},\;\kappa_{2}=1+a_{k}+c_{k}\\ \kappa_{3}= & 1+b_{k}^{-1}+c_{k}^{-1},\kappa_{4}=1+d_{k}^{-1} \end{array}$$

**Proof.**  In view of the triggering condition (6), one has

(17) $$\varepsilon_{k}\varepsilon_{k}^{T}\leq \varepsilon_{k}^{T}\varepsilon_{k}I\leq \tau^{2}I.$$

Using Lemma 2, we obtain

(18) $$-\varepsilon_{k}\vartheta^{T}\left(C_{k}x_{k}\right)-\vartheta \left(C_{k}x_{k}\right)\varepsilon_{k}^{T}\leq a_{k}\vartheta \left(C_{k}x_{k}\right)\vartheta^{T}\left(C_{k}x_{k}\right)+a_{k}^{-1}\varepsilon_{k}\varepsilon_{k}^{T},$$

(19) $$\varepsilon_{k}{\hat{x}}_{k}^{T}C_{k}^{T}+C_{k}{\hat{x}}_{k}\varepsilon_{k}^{T}\leq b_{k}\varepsilon_{k}\varepsilon_{k}^{T}+b_{k}^{-1}C_{k}{\hat{x}}_{k}{\hat{x}}_{k}^{T}C_{k}^{T},$$

(20) $$-\vartheta \left(C_{k}x_{k}\right){\hat{x}}_{k}^{T}C_{k}^{T}-C_{k}{\hat{x}}_{k}\vartheta^{T}\left(C_{k}x_{k}\right)\leq c_{k}\vartheta \left(C_{k}x_{k}\right)\vartheta^{T}\left(C_{k}x_{k}\right)+c_{k}^{-1}C_{k}{\hat{x}}_{k}{\hat{x}}_{k}^{T}C_{k}^{T},$$

and

(21) $$-\varepsilon_{k}v_{k}^{T}-v_{k}\varepsilon_{k}^{T}\leq d_{k}\varepsilon_{k}\varepsilon_{k}^{T}+d_{k}^{-1}v_{k}v_{k}^{T}.$$

Moreover, from the definition of the saturation function, we have

(22) $$E\left\{\vartheta \left(C_{k}x_{k}\right)\vartheta^{T}\left(C_{k}x_{k}\right)\right\}\leq \sum_{i=1}^{n_{y}}\varrho_{i}^{2}I.$$

It then follows from (13), (17)–(22) that

(23) $$\Lambda_{k}\leq {\bar{\Lambda }}_{k}.$$

Finally, considering (13), (15) and (23), we have $P_{k}^{f}\leq \Sigma_{k}^{f}$, which ends this proof. □

By the results obtained in Theorem 1, the following theorem is going to design a gain matrix $L_{k}$ such that the upper bound on the fault estimation error variance is minimized at each iteration.

**Theorem** **2.** *Under the constraint condition $L_{k}D_{k}=I$ and supposing that $a_{k}$, $b_{k}$, $c_{k}$ and $d_{k}$ are given positive scalars, the upper bound $\Sigma_{k}^{f}$ on the fault estimation error covariance is minimized, if the estimator gain $L_{k}$ is chosen as*

(24) $$L_{k}^{*}={\left(D_{k}^{T}{\bar{\Lambda }}_{k}^{-1}D_{k}\right)}^{-1}D_{k}^{T}{\bar{\Lambda }}_{k}^{-1}.$$

*Meanwhile, the minimum upper bound is given by*

(25) $$\Sigma_{k}^{f^{*}}={\left(D_{k}^{T}{\bar{\Lambda }}_{k}^{-1}D_{k}\right)}^{-1}.$$

**Proof.**  This proof is substantially to solve the following constrained optimization problem

(26) $$\begin{array}{cc} \min_{L_{k}} & \;\{\Sigma_{k}^{f}\}\\ s.t. & \;L_{k}D_{k}=I. \end{array}$$

By means of Lagrange multiplier method, we introduce the following Lagrange function

(27) $$\begin{array}{cc} \Xi (L_{k},\Upsilon_{k})= & L_{k}{\bar{\Lambda }}_{k}{L_{k}}^{T}+\left(I-L_{k}D_{k}\right)\Upsilon_{k}+\Upsilon_{k}^{T}{\left(I-L_{k}D_{k}\right)}^{T} \end{array}$$

where $\Upsilon_{k}$ is the Lagrange factor.

The derivatives of $\Xi (L_{k},\Upsilon_{k})$ with respect to $L_{k}$ and $\Upsilon_{k}$ can be written as

$$\frac{\partial \Xi (L_{k},\Upsilon_{k})}{\partial L_{k}}=2L_{k}{\bar{\Lambda }}_{k}-2\Upsilon_{k}^{T}D_{k}^{T}$$

and

$$\frac{\partial \Xi (L_{k},\Upsilon_{k})}{\partial \Upsilon_{k}}=2\left(I-L_{k}D_{k}\right).$$

Letting the above derivatives be zero, we have

(28) $$L_{k}^{*}=\Upsilon_{k}^{T}D_{k}^{T}{\bar{\Lambda }}_{k}^{-1}$$

and

(29) $$L_{k}^{*}D_{k}=I.$$

Then, substituting (28) into (29), one has

(30) $$\Upsilon_{k}={\left(D_{k}^{T}{\bar{\Lambda }}_{k}^{-1}D_{k}\right)}^{-1}$$

from which we have $L_{k}^{*}={\left(D_{k}^{T}{\bar{\Lambda }}_{k}^{-1}D_{k}\right)}^{-1}D_{k}^{T}{\bar{\Lambda }}_{k}^{-1}$.

Combining (27), (28) and (30), we derive the minimized upper bound on the fault estimation error covariance as follows

(31) $${\Sigma_{k}^{f}}^{*}=\Xi \left(L_{k}^{*},\Upsilon_{k}\right)={\left(D_{k}^{T}{\bar{\Lambda }}_{k}^{-1}D_{k}\right)}^{-1}.$$

The proof is now complete. □

### 3.2. State Estimation

In the following theorem, an upper bound on the state estimation error covariance is derived by means of the recursion and then minimized by the designed gain parameter $G_{k}^{*}$.

**Theorem** **3.** *Consider the state estimation error covariance obtained in (14). Assume that the condition $L_{k}D_{k}=I$ is satisfied and $\pi_{i}>0$(i=1,2,⋯,11) are arbitrary positive scalars. If matrix $\Sigma_{k}$ satisfies the following difference equation*

(32) $$\begin{array}{cc} \Sigma_{k+1}^{x}= & \phi_{1}\nu_{k}\nu_{k}^{T}\mathrm{tr}\left(\Sigma_{k}^{x}\right)+R_{k}+\phi_{2}\left(B_{k}-G_{k}D_{k}\right)\Sigma_{k}^{f}{\left(B_{k}-G_{k}D_{k}\right)}^{T}+\phi_{3}\tau^{2}G_{k}G_{k}^{T}\\ \; & +\phi_{4}\sum_{i=1}^{n_{y}}\varrho_{i}^{2}G_{k}G_{k}^{T}+\phi_{6}G_{k}Q_{k}G_{k}^{T}+\phi_{5}G_{k}C_{k}{\hat{x}}_{k}{\hat{x}}_{k}^{T}C_{k}^{T}G_{k}^{T}, \end{array}$$

*with the initial value $P_{0}^{x}\leq \Sigma_{0}^{x}$, where*

(33) $$\begin{array}{cc} \phi_{1}= & 1+\pi_{1}+\pi_{2}+\pi_{3}+\pi_{4},\;\phi_{2}=1+\pi_{2}^{-1}+\pi_{5}+\pi_{6}+\pi_{7},\\ \phi_{3}= & 1+\pi_{4}^{-1}+\pi_{6}^{-1}+\pi_{9}^{-1}+\pi_{10}^{-1}+\pi_{11}^{-1},\;\phi_{4}=1+\pi_{1}^{-1}+\pi_{7}^{-1}+\pi_{8}+\pi_{9},\\ \phi_{5}= & 1+\pi_{3}^{-1}+\pi_{5}^{-1}+\pi_{8}^{-1}+\pi_{10},\;\phi_{6}=1+\pi_{11}, \end{array}$$

*then $\Sigma_{k+1}^{x}$ is the upper bound of $P_{k+1}^{x}$, i.e., $P_{k+1}^{x}\leq \Sigma_{k+1}^{x}$.*

*Moreover, if the gain matrix $G_{k}$ is selected by*

(34) $$\begin{array}{cc} G_{k}^{*}= & \Phi_{k}\Psi_{k}^{-1} \end{array}$$

*where*

$$\Phi_{k}=\phi_{2}B_{k}\Sigma_{k}^{f}D_{k}^{T}$$

*and*

$$\begin{array}{cc} \Psi_{k}= & \phi_{2}D_{k}\Sigma_{k}^{f}D_{k}^{T}+\phi_{3}\tau^{2}I+\phi_{4}\sum_{i=1}^{n_{y}}\varrho_{i}^{2}I+\phi_{5}C_{k}{\hat{x}}_{k}{\hat{x}}_{k}^{T}C_{k}^{T}+\phi_{6}Q_{k}, \end{array}$$

*then the upper bound $\Sigma_{k+1}^{x}$ is minimized and the minimum upper bound is given by*

(35) $$\begin{array}{cc} \Sigma_{k+1}^{x^{*}}= & \phi_{1}\nu_{k}\nu_{k}^{T}\mathrm{tr}\left(\Sigma_{k}^{x}\right)+\phi_{2}B_{k}\Sigma_{k}^{f}B_{k}^{T}+R_{k}-\left\{\phi_{2}B_{k}\Sigma_{k}^{f}D_{k}^{T}\left\{\phi_{2}D_{k}\Sigma_{k}^{f}D_{k}^{T}+\phi_{3}\tau^{2}I\right.\right.\\ \; & +\left.{\left.\phi_{4}\sum_{i=1}^{n_{y}}\varrho_{i}^{2}I+\phi_{5}C_{k}{\hat{x}}_{k}{\hat{x}}_{k}^{T}C_{k}^{T}+\phi_{6}Q_{k}\right\}}^{-1}\right\}\left\{\phi_{2}B_{k}\Sigma_{k}^{f}D_{k}^{T}\left\{\phi_{2}D_{k}\Sigma_{k}^{f}D_{k}^{T}+\phi_{3}\tau^{2}I\right.\right.\\ \; & +{\left.{\left.\phi_{4}\sum_{i=1}^{n_{y}}\varrho_{i}^{2}I+\phi_{5}C_{k}{\hat{x}}_{k}{\hat{x}}_{k}^{T}C_{k}^{T}+\phi_{6}Q_{k}\right\}}^{-1}\right\}}^{T}. \end{array}$$

**Proof.**  By noting (14) and using Lemma 2, one has

(36) $$\begin{array}{cc} -{ℜ}_{k,1}-{ℜ}_{k,1}^{T}\leq & \pi_{1}E\left\{\left(h_{k}\left(x_{k}\right)-h_{k}\left({\hat{x}}_{k}\right)\right){\left(h_{k}\left(x_{k}\right)-h_{k}\left({\hat{x}}_{k}\right)\right)}^{T}\right\}+\pi_{1}^{-1}G_{k}E\left\{\vartheta \left(C_{k}x_{k}\right)\vartheta^{T}\left(C_{k}x_{k}\right)\right\}G_{k}^{T},\\ {ℜ}_{k,2}+{ℜ}_{k,2}^{T}\leq & \pi_{2}E\left\{\left(h_{k}\left(x_{k}\right)-h_{k}\left({\hat{x}}_{k}\right)\right){\left(h_{k}\left(x_{k}\right)-h_{k}\left({\hat{x}}_{k}\right)\right)}^{T}\right\}+\pi_{2}^{-1}\left(B_{k}-G_{k}D_{k}\right)P_{k}^{f}{\left(B_{k}-G_{k}D_{k}\right)}^{T},\\ {ℜ}_{k,3}+{ℜ}_{k,3}^{T}\leq & \pi_{3}E\left\{\left(h_{k}\left(x_{k}\right)-h_{k}\left({\hat{x}}_{k}\right)\right){\left(h_{k}\left(x_{k}\right)-h_{k}\left({\hat{x}}_{k}\right)\right)}^{T}\right\}+\pi_{3}^{-1}G_{k}C_{k}{\hat{x}}_{k}{\hat{x}}_{k}^{T}C_{k}^{T}G_{k}^{T},\\ {ℜ}_{k,4}+{ℜ}_{k,4}^{T}\leq & \pi_{4}E\left\{\left(h_{k}\left(x_{k}\right)-h_{k}\left({\hat{x}}_{k}\right)\right){\left(h_{k}\left(x_{k}\right)-h_{k}\left({\hat{x}}_{k}\right)\right)}^{T}\right\}+\pi_{4}^{-1}G_{k}E\left\{\varepsilon_{k}\varepsilon_{k}^{T}\right\}G_{k}^{T},\\ {ℜ}_{k,5}+{ℜ}_{k,5}^{T}\leq & \pi_{5}\left(B_{k}-G_{k}D_{k}\right)P_{k}^{f}{\left(B_{k}-G_{k}D_{k}\right)}^{T}+\pi_{5}^{-1}G_{k}C_{k}{\hat{x}}_{k}{\hat{x}}_{k}^{T}C_{k}^{T}G_{k}^{T},\\ {ℜ}_{k,6}+{ℜ}_{k,6}^{T}\leq & \pi_{6}\left(B_{k}-G_{k}D_{k}\right)P_{k}^{f}{\left(B_{k}-G_{k}D_{k}\right)}^{T}+\pi_{6}^{-1}G_{k}E\left\{\varepsilon_{k}\varepsilon_{k}^{T}\right\}G_{k}^{T},\\ -{ℜ}_{k,7}-{ℜ}_{k,7}^{T}\leq & \pi_{7}\left(B_{k}-G_{k}D_{k}\right)P_{k}^{f}{\left(B_{k}-G_{k}D_{k}\right)}^{T}+\pi_{7}^{-1}G_{k}E\left\{\vartheta \left(C_{k}x_{k}\right)\vartheta^{T}\left(C_{k}x_{k}\right)\right\}G_{k}^{T},\\ -{ℜ}_{k,8}-{ℜ}_{k,8}^{T}\leq & \pi_{8}G_{k}E\left\{\vartheta \left(C_{k}x_{k}\right)\vartheta^{T}\left(C_{k}x_{k}\right)\right\}G_{k}^{T}+\pi_{8}^{-1}G_{k}C_{k}{\hat{x}}_{k}{\hat{x}}_{k}^{T}C_{k}^{T}G_{k}^{T},\\ -{ℜ}_{k,9}-{ℜ}_{k,9}^{T}\leq & \pi_{9}G_{k}E\left\{\vartheta \left(C_{k}x_{k}\right)\vartheta^{T}\left(C_{k}x_{k}\right)\right\}G_{k}^{T}+\pi_{9}^{-1}G_{k}E\left\{\varepsilon_{k}\varepsilon_{k}^{T}\right\}G_{k}^{T},\\ {ℜ}_{k,10}+{ℜ}_{k,10}^{T}\leq & \pi_{10}G_{k}C_{k}{\hat{x}}_{k}{\hat{x}}_{k}^{T}C_{k}^{T}G_{k}^{T}+\pi_{10}^{-1}G_{k}E\left\{\varepsilon_{k}\varepsilon_{k}^{T}\right\}G_{k}^{T},\\ -{ℜ}_{k,11}-{ℜ}_{k,11}^{T}\leq & \pi_{11}G_{k}Q_{k}G_{k}^{T}+\pi_{11}^{-1}G_{k}E\left\{\varepsilon_{k}\varepsilon_{k}^{T}\right\}G_{k}^{T}. \end{array}$$

Combining (14), (33) and (36), we have

(37) $$\begin{array}{cc} P_{k+1}^{x}\leq & \phi_{1}E\left\{\left(h_{k}\left(x_{k}\right)-h_{k}\left({\hat{x}}_{k}\right)\right){\left(h_{k}\left(x_{k}\right)-h_{k}\left({\hat{x}}_{k}\right)\right)}^{T}\right\}+\phi_{2}\left(B_{k}-G_{k}D_{k}\right)P_{k}^{f}{\left(B_{k}-G_{k}D_{k}\right)}^{T}\\ \; & +\phi_{3}G_{k}E\left\{\varepsilon_{k}\varepsilon_{k}^{T}\right\}G_{k}^{T}+\phi_{4}G_{k}E\left\{\vartheta \left(C_{k}x_{k}\right)\vartheta^{T}\left(C_{k}x_{k}\right)\right\}G_{k}^{T}\\ \; & +\phi_{5}G_{k}C_{k}{\hat{x}}_{k}{\hat{x}}_{k}^{T}C_{k}^{T}G_{k}^{T}+\phi_{6}G_{k}Q_{k}G_{k}^{T}+R_{k}. \end{array}$$

From (3), (17), (22) and (37), we obtain

(38) $$\begin{array}{cc} P_{k+1}^{x}\leq & \phi_{1}\nu_{k}\nu_{k}^{T}\mathrm{tr}\left(P_{k}^{x}\right)+R_{k}+\phi_{2}\left(B_{k}-G_{k}D_{k}\right)P_{k}^{f}{\left(B_{k}-G_{k}D_{k}\right)}^{T}+\phi_{3}\tau^{2}G_{k}G_{k}^{T}\\ \; & +\phi_{4}\sum_{i=1}^{n_{y}}\varrho_{i}^{2}G_{k}G_{k}^{T}+\phi_{5}G_{k}C_{k}{\hat{x}}_{k}{\hat{x}}_{k}^{T}C_{k}^{T}G_{k}^{T}+\phi_{6}G_{k}Q_{k}G_{k}^{T}. \end{array}$$

By using Lemma 1, we arrive at $P_{k+1}^{x}\leq \Sigma_{k+1}^{x}$.

Next, the gain parameter $G_{k}$ given by (34) is ready to be optimal in the sense of minimizing the upper bound $\Sigma_{k+1}^{x}$.

The derivative of $\Sigma_{k+1}^{x}$ with respect to $G_{k}$ is computed by

(39) $$\begin{array}{cc} \frac{\partial }{\partial G_{k}}\Sigma_{k+1}^{x}= & -2\phi_{2}\left(B_{k}-G_{k}D_{k}\right)\Sigma_{k}^{f}D_{k}^{T}+2\phi_{3}\tau^{2}G_{k}+2\phi_{4}\sum_{i=1}^{n_{y}}\varrho_{i}^{2}G_{k}\\ \; & +2\phi_{5}G_{k}C_{k}{\hat{x}}_{k}{\hat{x}}_{k}^{T}C_{k}^{T}+2\phi_{6}G_{k}Q_{k}. \end{array}$$

Letting the derivative in (39) be zero, one has

(40) $$\begin{array}{cc} G_{k}^{*}= & \phi_{2}B_{k}\Sigma_{k}^{f}D_{k}^{T}{\left\{\phi_{2}D_{k}\Sigma_{k}^{f}D_{k}^{T}+\phi_{3}\tau^{2}I+\phi_{4}\sum_{i=1}^{n_{y}}\varrho_{i}^{2}I+\phi_{5}C_{k}{\hat{x}}_{k}{\hat{x}}_{k}^{T}C_{k}^{T}+\phi_{6}Q_{k}\right\}}^{-1}, \end{array}$$

and the minimum upper bound is given as the form as (35). The proof is now complete. □

Based on the above results, the developed ETSFE algorithm is summarized as follows (Algorithm 1).
**Algorithm 1:** ETSFE algorithm1. Let parameters $a_{k}$, $b_{k}$, $c_{k}$, $d_{k}$, $\pi_{i}\left(i=1,2,\cdots ,11\right)$ be given. Set initial values ${\hat{x}}_{0}={\bar{x}}_{0}$ and $\Sigma_{0}^{x}=P_{0}^{x}$, the
length of time horizon *N* and k=0;2. Calculate the fault estimator gain matrix $L_{k}^{*}$ according to (24), the upper bound of the fault estimation
error covariance ${\Sigma_{k}^{f}}^{*}$ via (25), and the fault estimate ${\hat{f}}_{k}$ according to (7);3. Calculate the state estimator gain matrix $G_{k}^{*}$ according to (34), the upper bound of the state estimation
error covariance ${\Sigma_{k}^{x}}^{*}$ via (35), and the state estimate ${\hat{x}}_{k}$ according to (7);4. If k≤N, set k=k+1 and go to step 2, else go to step 5;5. Stop.

**Remark** **2.** *Theorems 2 and 3 provide the expressions for the estimator gain matrices $L_{k}$ and $G_{k}$, respectively, and the desired estimator has been designed. In the design of the estimator, three difficulties can be identified as follows: (1) how can we choose an appropriate standard to evaluate the estimation performance when the systems are subject to nonlinearities and sensor saturations under the event-triggering protocol? (2) how can we construct an appropriate estimator structure which is closely related to the selection of the estimation analysis method? (3) After the estimator structure is determined, how can we obtain the expressions for the estimator gain matrices by using the mathematical tool appropriately. In this paper, these questions have been well answered.*

**Remark** **3.** To date, we have studied the ETSFE problems for nonlinear systems subject to sensor saturations. Relying on the matrix analysis technique, the estimation error covariances for the state and fault are both guaranteed by upper bounds, and then such upper bounds have been minimized by appropriately designing the gain parameters $L_{k}^{*}$ and $G_{k}^{*}$. Comparing with the existing methods in literature [10,21], the estimation approach proposed in this paper is capable of dealing with state and fault simultaneous estimation problem when the systems are subject to the nonlinearities including the sensor saturation nonlinearity under the event-triggering protocols. Actually, the proposed estimation approach is applicable to all those situations where the complex phenomena could be modelled by a bound-limited variable. Moreover, the corresponding ETSFE algorithm proposed is of a simple and recursive form, which is suitable for online computation. The following section will provide two illustrative examples to validate the usefulness of the developed ETSFE algorithm.

## 4. Experimental Simulation

In this section, two simulation examples are utilized to validate the usefulness of the developed ETSFE algorithm.

**Example** **1.** *The system under consideration in (1) has the following parameters*

(41) $$\begin{array}{cc} A_{k}= & \left[\begin{array}{cc} 0.6 & -0.5\\ 0.2 & 0.4+0.4\cos(k) \end{array}\right],\;B_{k}={\left[0.9\;-0.7\right]}^{T},\\ C_{k}= & \left[\begin{array}{cc} -0.4 & 1\\ -0.3 & 1.5 \end{array}\right],\;D_{k}={\left[-0.9\;0.9\right]}^{T},\;\nu_{k}=0.15. \end{array}$$

*Let the nonlinear function $h_{k}\left(x_{k}\right)$ be*

(42) $$h_{k}\left(x_{k}\right)=A_{k}x_{k}+{\bar{h}}_{k}\left(x_{k}\right)$$

*where ${\bar{h}}_{k}\left(x_{k}\right)={\left[\begin{array}{cc} 0.17\sin(x_{k}^{1}) & 0.16\sin(x_{k}^{2}) \end{array}\right]}^{T}$.*

The process noise $w_{k}$ and measurement noise $v_{k}$ are Gaussian noises with zero mean and their covariances are set as $R_{k}=\left[\begin{array}{cc} 0.3 & 0\\ 0 & 0.3 \end{array}\right]$ and $Q_{k}=\left[\begin{array}{cc} 0.2 & 0\\ 0 & 0.2 \end{array}\right]$, respectively. The saturation levels are $\varrho_{1}=\varrho_{2}=0.1$ and the threshold of the triggering threshold is τ=1.2. In this example, the parameters $\pi_{i}$(i=1,2,…,11), $a_{k}$, $b_{k}$, $c_{k}$ and $d_{k}$ are chosen as 1, and the initial state is chosen as $x_{0}={\left[0.5\;-0.5\right]}^{T}$. Based on the above parameters, the gain matrices $L_{k}^{*}$ and $G_{k}^{*}$ can be iteratively computed by (24) and (34).

**Figure 2:**
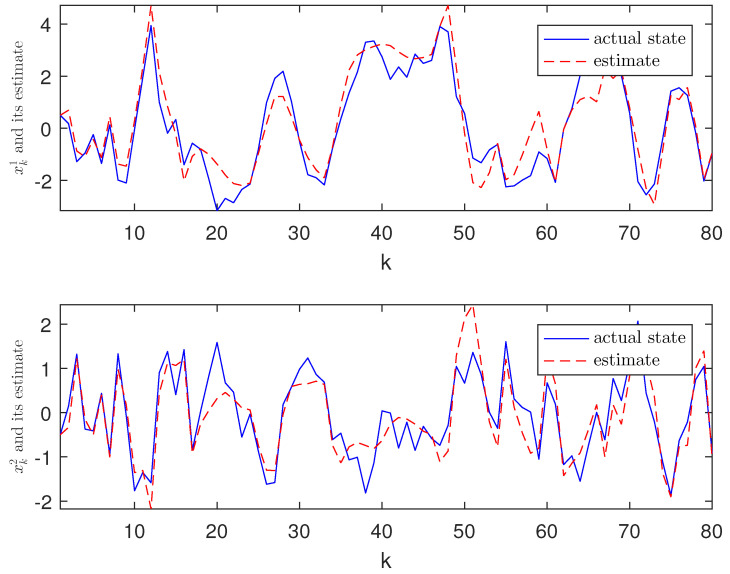
Trajectories $x_{k}^{1}$, $x_{k}^{2}$ and their estimates.

**Figure 3:**
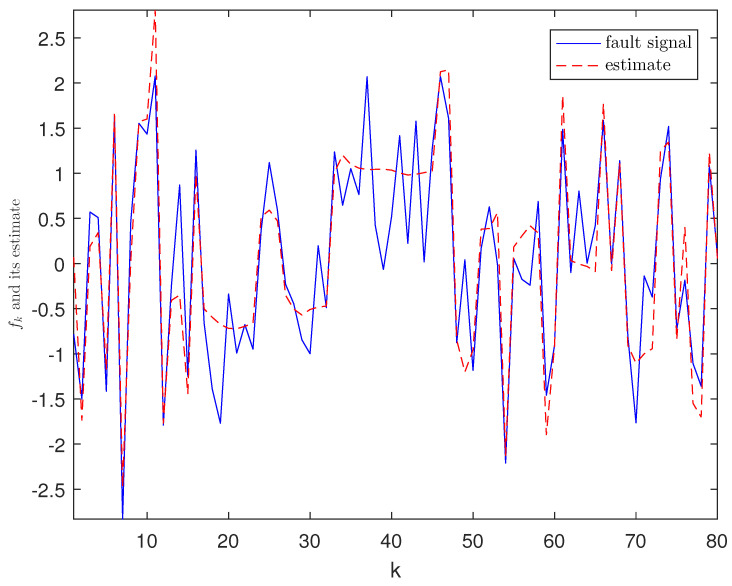
Trajectories of fault $f_{k}$ and its estimate.

**Figure 4:**
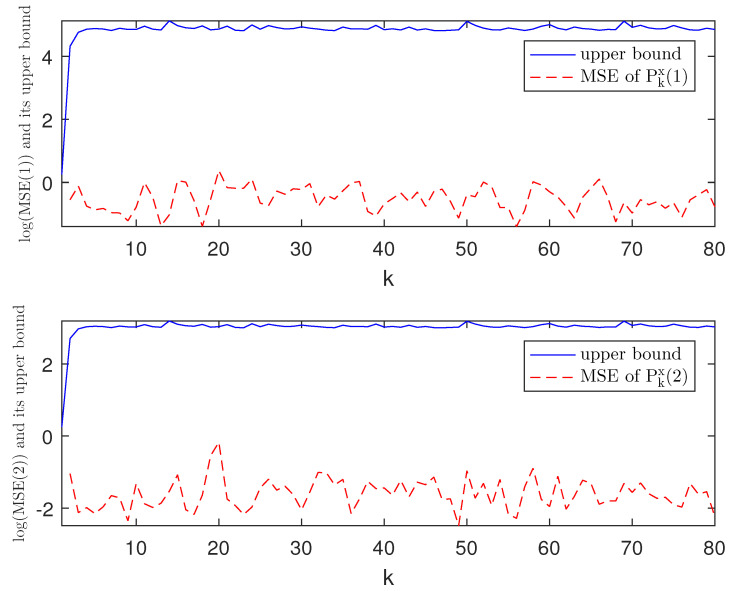
$\mathrm{Log}({\mathrm{MSE}}_{k}^{x})$ of $x_{k}^{1}$, $x_{k}^{2}$ and the traces of their upper bounds.

**Figure 5:**
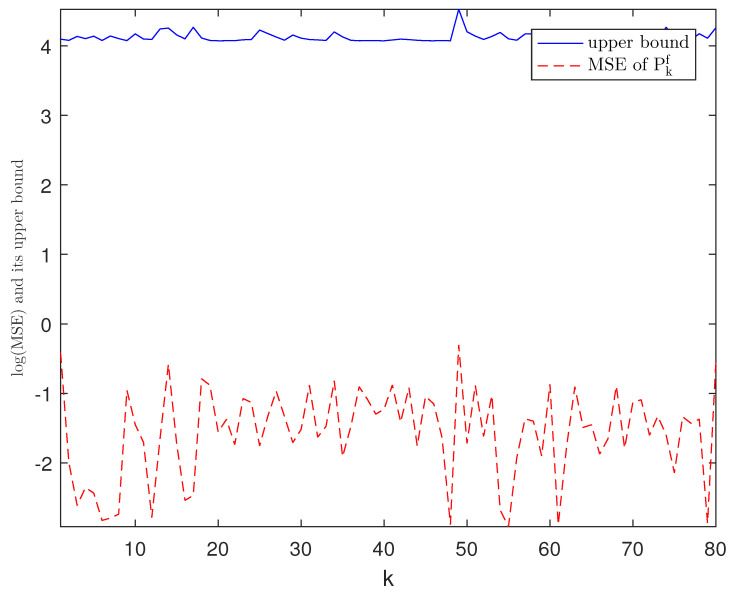
$\mathrm{Log}({\mathrm{MSE}}_{k}^{f})$ of $f_{k}$ and the trace of its upper bound.

*The designed algorithm is validated in MATLAB (R2016a), Intel Core CPU i5-8265. Figure 2, Figure 3, Figure 4 and Figure 5 show the simulation results based on the parameters mentioned above. The above picture in Figure 2 plots the state trajectory and its estimate for $x_{k}^{1}$ and the picture below shows the state trajectory and the estimate for $x_{k}^{2}$. The fault signals as well as its estimates are shown in Figure 3. It is seen from Figure 2 and Figure 3 that the proposed estimator performs well. The mean square errors (${\mathrm{MSE}}_{k}$) and the traces of their minimal upper bounds for state are shown in Figure 4. The corresponding results for fault are given in Figure 5. The simulation results shown in Figure 4 and Figure 5 concur with our theoretical analysis.*

**Remark** **4.** *Due to the effects of the nonlinearities and the event-triggering protocol, upper bounds of the estimation error covariances are employed as the performance index of state and fault estimation. Such a practice inevitably gives rise to conservatism which may affect the real estimation accuracy. Although the estimator gains are designed to minimize the upper bounds at each step, the minimized upper bounds are actually not tight. Note from Theorems 2 and 3 that the minimized upper bounds are closely related to the parameters $a_{k}$, $b_{k}$, $c_{k}$, $d_{k}$, $\pi_{i}>0$(i=1,2,⋯,11). Therefore, in the experiment, these parameters should be selected prudently and the appropriate selection of these parameters may further improve the experimental results.*

**Example** **2.** *In this experiment, a ballistic object tracking example is employed to further validate the feasibility of the proposed ETSFE algorithm. The ballistic object tracking system is described by [21,32]*

$$\begin{array}{cc} x_{k+1}= & h_{k}\left(x_{k}\right)+B_{k}f_{k}+w_{k}\\ y_{k}= & \vartheta \left(C_{k}x_{k}\right)+D_{k}f_{k}+v_{k} \end{array}$$

*where $h_{k}\left(x_{k}\right)=A_{k}x_{k}+{\bar{h}}_{k}\left(x_{k}\right)$, ${\bar{h}}_{k}\left(x_{k}\right)=\nu_{k}\left(g_{k}\left(x_{k}\right)+G\right)$, $g_{k}\left(x_{k}\right)=-\frac{g\psi (x_{2,k})}{2\Upsilon }\sqrt{{\dot{x}}_{1,k}^{2}+{\dot{x}}_{2,k}^{2}}\left[\begin{array}{c} {\dot{x}}_{1,k}\\ {\dot{x}}_{2,k} \end{array}\right]$, $\psi \left(x_{2,k}\right)=\kappa_{1}\cdot \exp\left(-\kappa_{2}x_{2,k}\right)$ and*

$$\begin{array}{cc} A_{k}= & \left[\begin{array}{cccc} 1 & T & 0 & 0\\ 0 & 1 & 0 & 0\\ 0 & 0 & 1 & T\\ 0 & 0 & 0 & 1 \end{array}\right],\;B_{k}=\left[\begin{array}{c} 0.01\\ 0\\ 0.01\\ 0 \end{array}\right],\;C_{k}=\left[\begin{array}{cccc} 1 & 0 & 0 & 0\\ 0 & 0 & 1 & 0 \end{array}\right],\\ D_{k}= & \left[\begin{array}{c} 0.01\\ 0\\ 0.01\\ 0 \end{array}\right],\;G=\left[\begin{array}{c} 0\\ -g \end{array}\right],\;\nu_{k}=\left[\begin{array}{cc} \frac{T^{2}}{2} & 0\\ T & 0\\ 0 & \frac{T^{2}}{2}\\ 0 & T \end{array}\right]. \end{array}$$

*The parameters involved in the above system are stated as follows: $x_{k}={\left[x_{1,k}\;{\dot{x}}_{1,k}\;x_{2,k}\;{\dot{x}}_{2,k}\right]}^{T}$ where $x_{1,k}$, ${\dot{x}}_{1,k}$ represent respectively the position and the velocity of the target on x-axis and $x_{2,k}$, ${\dot{x}}_{2,k}$ represent the position and the velocity of the target on y-axis; T is the sampling period; g denotes the gravity acceleration; *Υ* represents the ballistic coefficient; the exponentially decaying function ψ(·) is the air density; and $w_{k}$, $v_{k}$ are both Gaussian noises with zero mean and their covariances are $R_{k}=\lambda \cdot \mathrm{diag}\left\{{\bar{R}}_{k},{\bar{R}}_{k}\right\}$ and $Q_{k}=10I_{2}$ where ${\bar{R}}_{k}=\left[\begin{array}{cc} \frac{T^{3}}{3} & \frac{T^{2}}{2}\\ \frac{T^{2}}{2} & T \end{array}\right]$ and λ is a parameter related to the process noise.*

*In this example, the triggering thresholds are set as $\tau_{1}=\tau_{2}=200$ and the saturation levels are selected by $\varrho_{1}=\varrho_{2}=6000$. The other parameters are chosen as g=9.8 m/s${}^{2}$, $\Upsilon =4\times 10^{4}$ kg/ms${}^{2}$, $\lambda =0.1\;m^{2}/s^{3}$, T=1 s, $\kappa_{1}=1.227$, $\kappa_{2}=1.094\times 10^{-4}$, $\pi_{i}=1$(i=1,2,…,11), $a_{k}=b_{k}=c_{k}=d_{k}=1$.*

**Figure 6:**
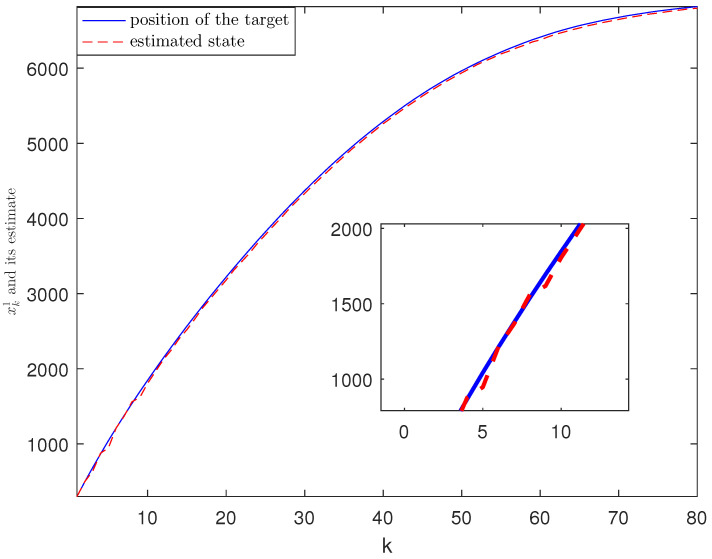
The position of the target $x_{1,k}$ and its estimate.

**Figure 7:**
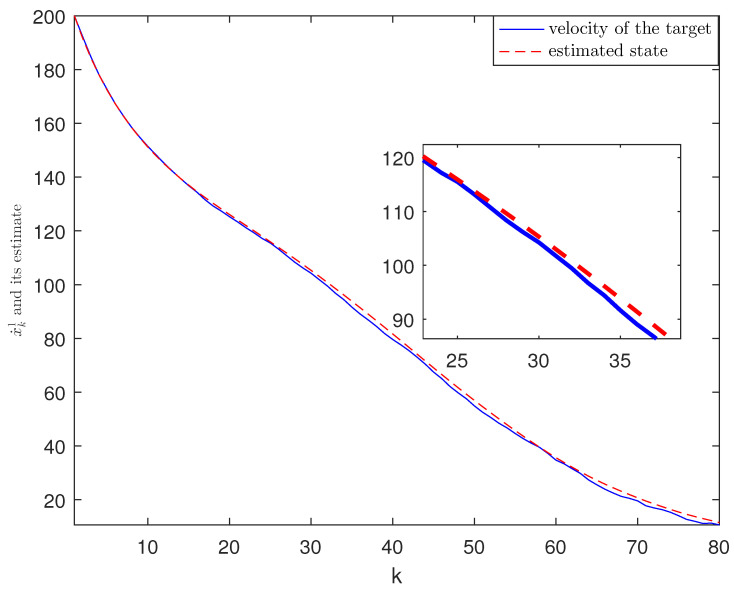
The velocity of the target ${\dot{x}}_{1,k}$ and its estimate.

**Figure 8:**
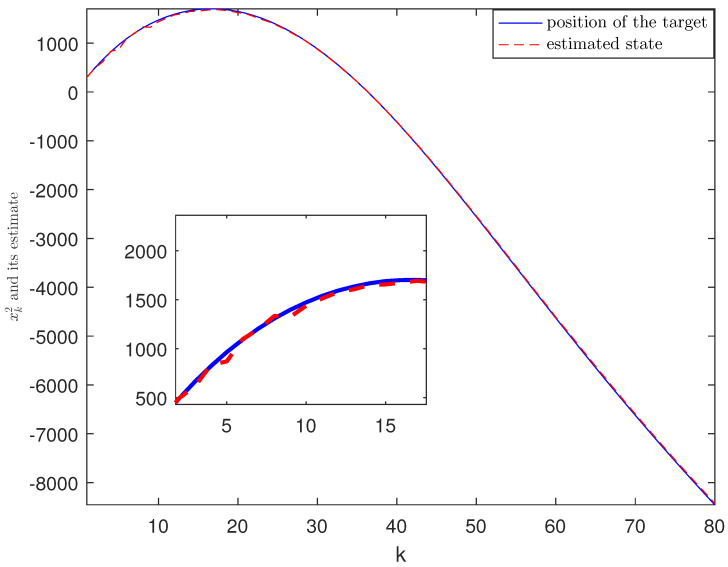
The position of the target $x_{2,k}$ and its estimate.

**Figure 9:**
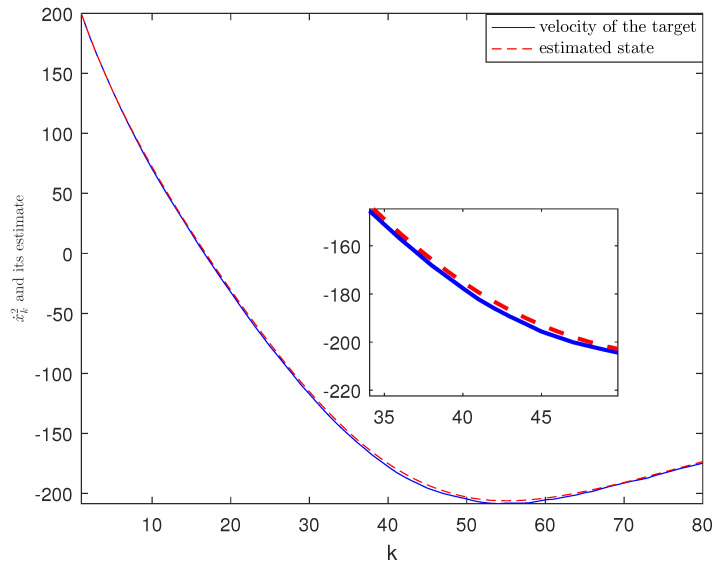
The velocity of the target ${\dot{x}}_{2,k}$ and its estimate.

**Figure 10:**
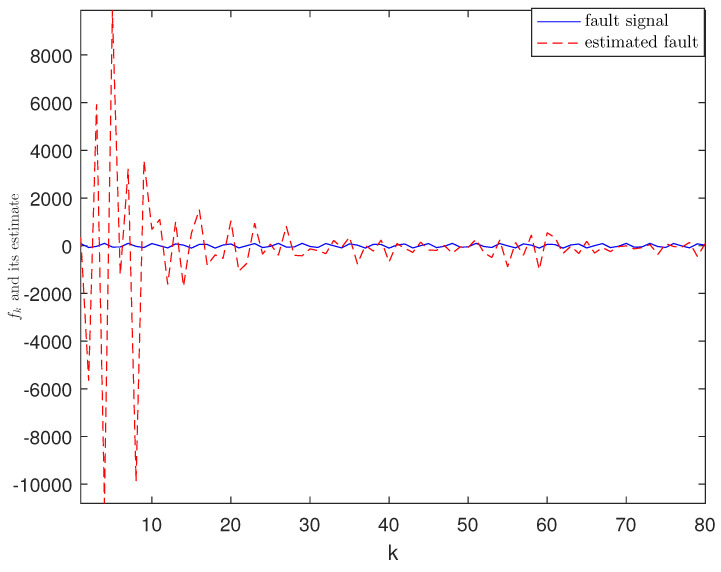
The actual fault and its estimate.

*The simulation results are displayed in Figure 6, Figure 7, Figure 8, Figure 9 and Figure 10. The position of the target on x-axis $x_{1,k}$ and its estimate are plotted in Figure 6. Figure 7 shows the velocity of the target on x-axis ${\dot{x}}_{1,k}$ and its estimate. The corresponding results for the target on y-axis are depicted in Figure 8 and Figure 9. The actual fault and its estimate are shown in Figure 10. From the simulation, it is observed that the proposed ETSFE algorithm is indeed effective in the ballistic object tracking system.*

## 5. Conclusions

In this paper, we have addressed the state and fault estimation issues for nonlinear systems subject to sensor saturations. The ETM based scheduling protocol has been adopted to manage the measurement transmission, and thus relieving the communication burden. Relying on the received measurements, the state and fault estimator has been designed where the recursions of the upper bounds on the estimation error covariances for the state and fault have been given, respectively. Then, the derived upper bounds have been minimized by properly designing the estimator gain matrices. Finally, we have utilized two simulation examples to show the validity of the designed ETSFE algorithm. Our future research topics would be to extend the main results in this paper to the sensor networks where multiple coupling sensors are involved [22] and to apply the main results in practical engineering such as power systems [3].

## Data Availability

Data sharing not applicable.
